# Developing a High-Throughput Platform for the Discovery of Sustainable Antibacterial Materials

**DOI:** 10.1021/acsami.4c14689

**Published:** 2024-10-25

**Authors:** Krzysztof Wieczerzak, Fedor F. Klimashin, Amit Sharma, Stefanie Altenried, Katharina Maniura-Weber, Qun Ren, Johann Michler

**Affiliations:** † Laboratory for Mechanics of Materials and Nanostructures, Empa, Swiss Federal Laboratories for Materials Science and Technology, CH-3602 Thun, Switzerland; ‡ Laboratory for Biointerfaces, Empa, Swiss Federal Laboratories for Materials Science and Technology, Lerchenfeldstrasse 5, CH-9014 St. Gallen, Switzerland

**Keywords:** material library, high-throughput characterization, sustainable antimicrobial surfaces, mechanical properties, healthcare-associated infections, antimicrobial resistance

## Abstract

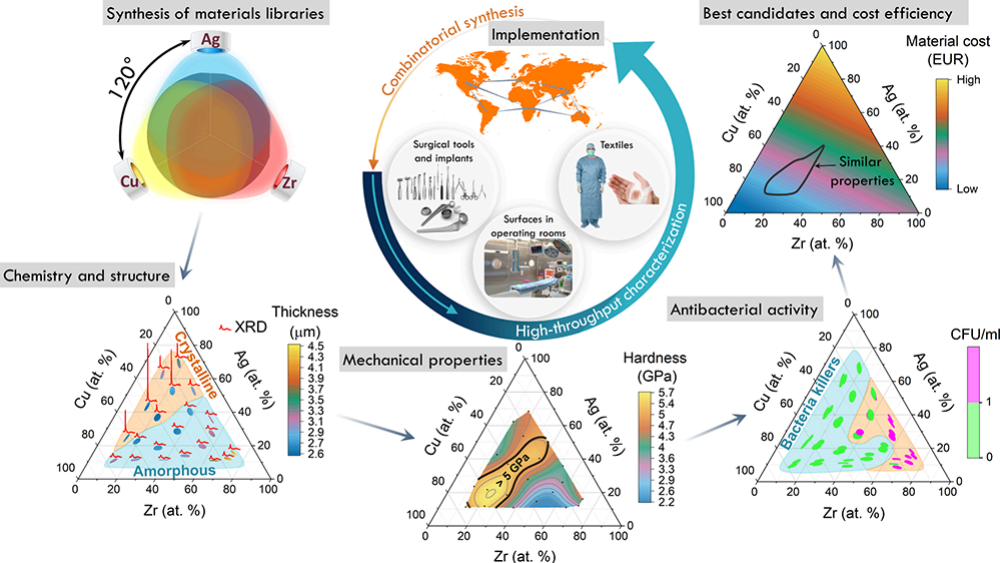

Healthcare-associated infections (HCAIs) pose a significant global health challenge, exacerbated by the rising threat of antimicrobial resistance (AMR). This study introduces a high-throughput platform designed to identify sustainable antibacterial surfaces, exemplified by a copper–silver–zirconium (CuAgZr) alloy library. Utilizing combinatorial synthesis and advanced characterization techniques, material libraries (MatLibs) are generated and evaluated to rapidly screen diverse alloy compositions. The results demonstrate the ability to reproducibly create alloys with significant antimicrobial properties and high hardness, making them suitable for biomedical applications. The study highlights the critical role of compositional precision in developing materials that balance mechanical strength with antibacterial efficacy. Additionally, this approach ensures significant cost-effectiveness, facilitating the identification of economically viable alloy compositions. This research underscores the potential of high-throughput materials science to expedite the discovery of sustainable solutions for reducing HCAIs and addressing AMR, signaling a leap forward in sustainable healthcare material development.

## Introduction

In the realm of global health, healthcare-associated infections (HCAIs) represent a formidable challenge, with surfaces in medical settings serving as potential conduits for the transmission of pathogenic microorganisms.^1^ In the United States, HCAIs were linked to approximately 99000 deaths in 2002, underscoring their severe health implications and the associated economic burden estimated at $6.5 billion annually by 2004.^2^ The European Centre for Disease Prevention and Control has similarly reported that around 8.9 million HCAIs occur yearly across European hospitals and long-term care facilities (LTCFs), highlighting a widespread issue that affects 1 in 15 hospital patients and 1 in 24 long-term care residents.^3^ In Switzerland in 2019, HCAIs (along with LTCF-acquired infections) accounted for 19.8% of the indications for antimicrobial use, following community-acquired infections at 45.9%, and surgical prophylaxis at 22.3%.^4^ A particularly alarming aspect of HCAIs is their contribution to the escalation of antimicrobial resistance (AMR), a global crisis that threatens to render current antibiotic treatments ineffective, with predictions suggesting AMR could lead to 10 million deaths annually by 2050 and potentially reduce global gross domestic product by up to $100 trillion.^5,6^

The significance of AMR is compounded by the fact that one out of every three bacteria responsible for infections in hospitals and LTCF is resistant to antibiotics, emphasizing the urgent need for innovative solutions.^3^ This challenge extends into the operating room, where surfaces often remain contaminated despite rigorous cleaning standards, serving as reservoirs for pathogens that can be transferred onto the hands of personnel and subsequently to patients, increasing the risk of HCAI and potential infection outbreaks.^7^

Given the complexity of this issue, our study focuses on developing a high-throughput platform to identify sustainable antibacterial surfaces that can significantly reduce HCAI incidence while also addressing AMR. Traditionally, metal-based materials such as stainless steels are commonly used for surgical instruments and some furniture in hospitals due to their corrosion resistance and durability.^8^ While stainless steels are durable, they do not inherently possess antibacterial properties, which necessitates additional coatings or treatments to enhance their antimicrobial efficacy.^9,10^

Here, the potential of a new high-throughput platform for discovering new materials is demonstrated using a copper–silver–zirconium (CuAgZr) alloy system. This system is chosen due to previously reported significant antimicrobial properties and hardness in some of its alloys, which are critical for biomedical applications.^11−13^ Both copper and silver are well-known for their antibacterial efficacy, with copper’s ability to induce bacterial membrane depolarization^14,15^ and silver’s well-documented role in disrupting bacterial cell walls.^14,16^ Recent studies, such as Sahin et al.,^17^ have also highlighted the additional potential of such metal-containing systems for applications beyond antibacterial effects, including their use in bacterial detection via surface-enhanced Raman scattering. These findings emphasize the versatility of Cu and Ag in biomedical contexts, strengthening the rationale for selecting this alloy system. The novel approach, proposed in this work, departs from traditional materials research and development by utilizing rapid assessment methodologies to screen material libraries (MatLibs) for desired traits.^18−20^ Our high-throughput methodology leverages the combinatorial synthesis of diverse alloy compositions, allowing for the rapid analysis and selection based on structural, mechanical, and antimicrobial characteristics. By accelerating the evaluation process, this method overcomes the financial and temporal constraints typically associated with the development of new materials, paving the way for more effective and timely solutions in the fight against HCAIs and AMR.

This study aims to demonstrate how advanced high-throughput materials science can rapidly identify and evaluate materials with dual capabilities: combating microbial threats and enduring the rigors of medical applications, thus embodying the essence of sustainability. Identifying alloys with a similar set of properties allows for further optimization, considering economic factors and environmental responsibility, such as selecting materials with a lower carbon footprint and that are easier to recycle. Choosing materials with enhanced durability that can maintain their antibacterial properties through prolonged use and repeated cleaning can be crucial for sustainable healthcare by reducing the incidence of HCAIs and addressing the growing issue of AMR. By focusing on the innovative assessment of the CuAgZr system, we contribute to broader efforts to combat HCAIs and AMR. In doing so, we highlight the importance of high-throughput materials science in offering sustainable solutions to one of the most pressing health crises of our time.^5,6^

## Results and Discussion

### Combinatorial Synthesis: A Path to Diversity in Alloy Design

**Figure 1:**
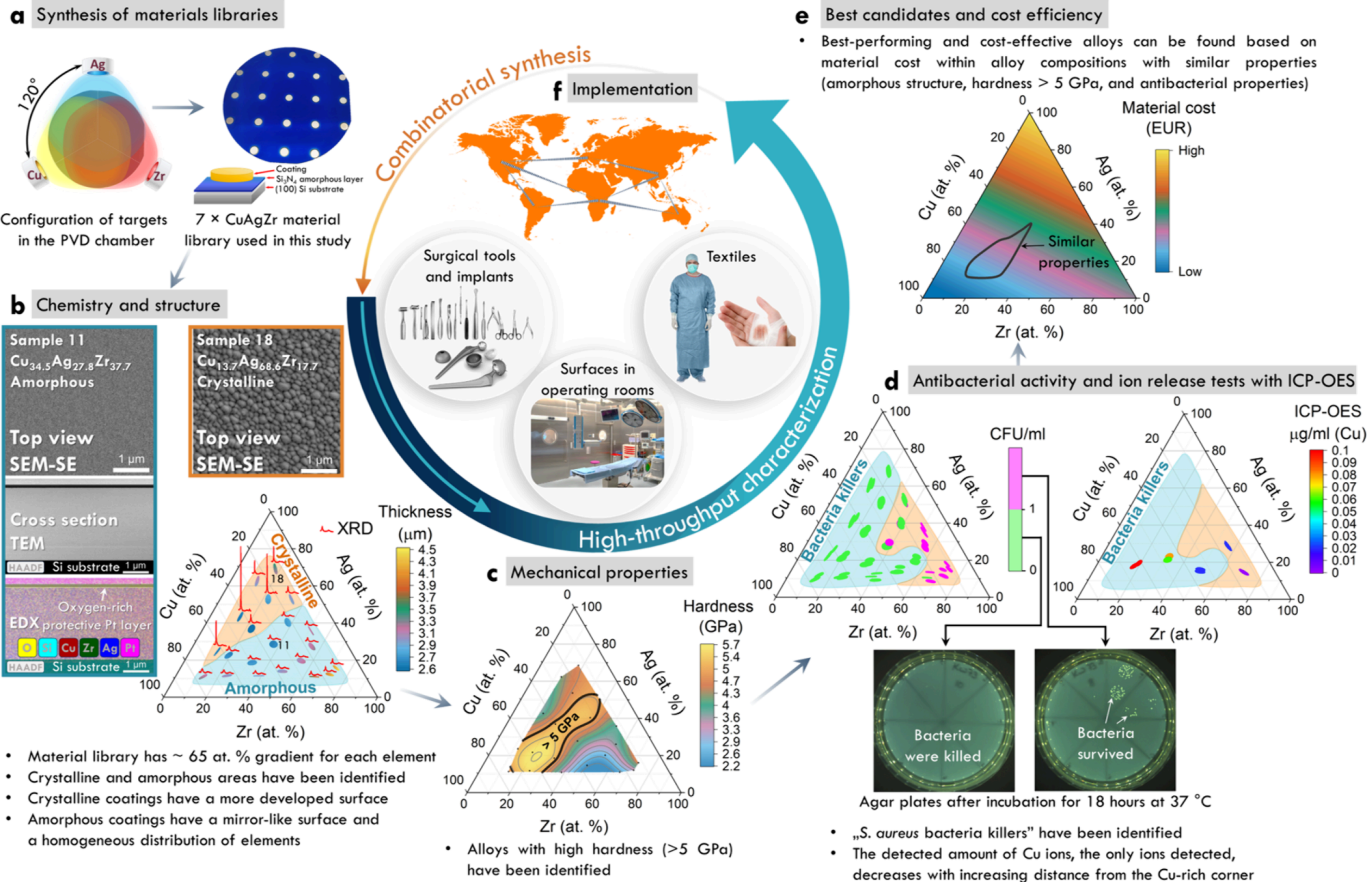
Combinatorial synthesis and high-throughput characterization of CuAgZr alloy libraries for biomedical applications. (a) Configuration of targets within the PVD chamber, leading to the creation of seven CuAgZr MatLibs with the same chemical gradient utilized in this study. The material library was created in the form of 21 circular patches with a diameter of 5 mm. (b) Results of chemical and microstructure analysis. The ellipses on the ternary system show the actual chemical composition gradient within each of the studied patches. The color corresponds to the thickness of the coating, indicating its differences depending on the position of a given area in relation to the magnetrons during synthesis. XRD analysis allowed for the identification of amorphous areas and crystalline ones with an FCC structure. Microscopic studies (SEM and TEM) and EDX revealed chemical and structural differences between amorphous and crystalline samples, emphasizing the morphology of the surface and the uniform distribution of elements in the amorphous sample (#11). (c) Correlation between the alloys’ chemical composition and their hardness. The contour lines and color gradient represent different levels of hardness, with the area labeled “>5 GPa” indicating regions of particularly high hardness. (d) Antibacterial efficacy assessed through agar plating assay and ion release tests with ICP-OES. The left ternary diagram shows areas where CuAgZr alloys successfully killed *S. aureus*, while the right diagram shows the copper ion concentration from the selected patches as detected by ICP-OES. The agar plates beneath demonstrate actual antibacterial results from selected patches. (e) Material cost-effectiveness plot identifying the most promising candidates for further development based on a balance between the structure, mechanical properties, antibacterial activity, and alloy cost. (f) Implementation of sustainable antibacterial materials. This graphic illustrates potential applications including coatings for surgical tools and implants, operating room surfaces, and antibacterial additives for textiles. The best alloys can be manufactured in bulk form for entire tools, implants, and furniture in operating rooms, indicating that material selection utilizing this technology is not limited to mere surface treatments. High-throughput combinatorial studies enhance market resilience to geopolitical shifts and related raw material access issues, allowing for the rapid substitution of elements if access becomes restricted.

In the quest for novel materials capable of meeting the stringent demands of biomedical applications, the combinatorial synthesis approach is an innovative strategy. In this work we employed a direct-current magnetron sputtering (DCMS), which is a form of physical vapor deposition (PVD).^21^ Cosputtering, the technique used in our study, involves the simultaneous sputtering of multiple targets. This process allows for the creation of alloy coatings with various elemental compositions on a single substrate (Figures 1a and S1). By adjusting the relative power supplied to each target and the distance of the substrate from the magnetron, the composition of the deposited materials can be precisely controlled. Thus, magnetron cosputtering provides an efficient pathway to explore a broad compositional space within a single experiment. This gradient approach is particularly potent for identifying regions within the compositional space that meet the desired balance of properties.^18−20^ A total of seven MatLibs were synthesized and subsequently utilized for the characterization of various properties, including structural characteristics through X-ray diffraction (XRD), mechanical properties via nanoindentation, surface morphology through scanning electron microscopy (SEM), studies of antibacterial properties, and ion release tests, demonstrating a comprehensive approach to material evaluation.

The meticulous application of calibrated combinatorial synthesis led to the creation of MatLibs, each achieving a compositional gradient of approximately 65 atom % for each element across a wide area of the CuAgZr ternary system, as shown in Figure 1b. The chemical composition of the MatLibs produced is also summarized in Tables S1–S3. The combinatorial synthesis enabled access to approximately 30000 distinct alloys, assuming a resolution of 1 atom % (a difference in the content of any given element between two alloys of 0.33 atom %). It is also worth noting that depending on the distance of a given patch from the magnetron, a significantly different chemical composition gradient was formed, which has been represented using ellipses on the ternary diagram. Patches located on the edges of the MatLib exhibit a chemical composition gradient in the shape of stretched ellipses, while patches located in the central part of the MatLib have a chemical composition gradient in a shape closer to circular (see also Figure S2). Understanding the implications of these compositional gradients is crucial, particularly when interpreting subsequent antibacterial studies performed on these surfaces.

### From Chemistry to Structure

Each patch underwent XRD analysis to elucidate its structure (Figures 1b and S3). The XRD studies were pivotal in demarcating the amorphous from the crystalline regions, the latter possessing a face-centered-cubic (FCC) structure. Based on the analysis of the diffractograms, it can be observed that near the Cu–Ag edge and the Ag-rich corner, crystalline alloys are predominantly present. As one moves on the ternary diagram toward the Zr-rich areas, there is an increase in the proportion of amorphous regions. The areas near the Cu–Zr edge are fully amorphous within the studied range of chemical compositions, while the regions near the Ag–Zr edge remain amorphous up to a Zr content between 50% and 60%. These observations indicate that adding Zr to Cu increases the tendency for glass formation to a greater extent than adding Zr to Ag.

A characteristic feature of the crystalline areas is their developed surface morphology, as opposed to the smooth finish prevalent in most amorphous regions’ distribution (Figures 1b and S4). The amorphous structure not only provides a smoother surface but also offers a homogeneous microstructure and element distribution, as demonstrated by the cross-sectional view of the amorphous Cu_34.5_Ag_27.8_Zr_37.7_ alloy in Figure 1b. The elemental map obtained using energy-dispersive X-ray spectroscopy (EDX) confirms the uniform distribution of alloying elements.

### Interplay of Composition and Mechanical Properties

The essence of the nanoindentation experiments lies in understanding how different combinations of copper, silver, and zirconium influence the hardness of the alloy, a characteristic that strongly correlates with a material’s ability to resist wear.^22^ What makes high hardness significant is its implication for longevity and sanitation in medical applications. Harder surfaces are less likely to scratch or degrade, reducing the likelihood of forming niches where bacteria can thrive. Moreover, the metallic components of these alloys, particularly copper and silver, are known for their antibacterial properties.^14,23−25^ When used as a coating, these materials could potentially reduce the risk of infection by creating an unfavorable surface for bacterial growth.

The impact of the chemical composition on hardness is illustrated in Figure 1c. The reduced modulus is shown in Figure S5. The results of mechanical properties are also compiled in Table S1. Figure 1c illustrates how the hardness of CuAgZr alloys varies with chemical composition, ranging broadly from 2.2 to 5.7 GPa. Interestingly, the hardest alloys have a higher copper content, while the softest are enriched in zirconium. This finding is counterintuitive since zirconium has the highest melting point among the elements in the CuAgZr system, typically associated with higher bond energy. However, upon analyzing the morphology of the films (Figure S4), it was found a distinct increase in cracks within the zirconium-enriched areas. These cracks undoubtedly affect the mechanical properties of the alloys, leading to a pronounced decrease in hardness.

### Fight against Bacteria with Novel Alloys

The antibacterial tests in this study involved incubating bacterial pathogen *Staphylococcus aureus* with CuAgZr MatLibs of different compositions to observe their antimicrobial effect. After a 2-h period, the bacteria that survived contact with the materials were transferred to agar plates and allowed to grow, with the results indicating how effective each material was at killing bacteria. It is important to note that *S. aureus* is a Gram-positive bacterium, which has a thicker peptidoglycan layer in its cell wall. Our study did not include testing on Gram-negative bacteria, which have a more complex cell wall structure with an additional outer membrane.^17^ This difference in cell wall architecture may influence the antibacterial performance of the materials, and testing against Gram-negative bacteria, such as *Escherichia coli*, could be an interesting direction for future research to provide a more comprehensive evaluation of the CuAgZr alloys’ antibacterial efficacy.

The ternary diagram shown in Figure 1d delineate the compositional spectrum of the alloys and their corresponding antibacterial efficacies. Antibacterial tests results are also summarized in Table S2. Regions where no viable bacteria were observed, indicated by green ellipses, point to compositions with strong antibacterial properties. These are observed in areas with higher contents of copper and/or silver. Moving toward the zirconium-rich corner, there is a noticeable decline in antibacterial effectiveness. This correlation is based on agar plating assay: bacteria that were in contact with alloys rich in copper and/or silver showed no regrowth on agar (all bacteria perished during exposure to the bactericidal surface), whereas those with zirconium-rich alloys resulted in the development of bacterial colonies on the agar plates.

To understand whether the release of metal ions from alloys affects their antibacterial properties, measurements were carried out on selected samples using inductively coupled plasma optical emission spectroscopy (ICP-OES). The results are also compiled in Table S3 and Figure S6. During the tests, only copper ions were detected. The results show that as the distance from the copper-rich corner increases, the amount of released copper ions decreases. In areas of the CuAgZr ternary system where no antibacterial properties were observed, no copper ions or only a very low concentration (0.02 μg/mL) were detected. It is not clear why no silver ions release could be detected. Thus, despite the well-known antibacterial properties of silver, it is difficult to assess its exact role in the studied alloys.

### Insights from High-Throughput Synthesis and Characterization

The results of this work confirm the crucial role of compositional precision in developing antibacterial alloys for biomedical applications. Chemical-composition-dependent and structure-dependent differences in surface morphology are expected to play a significant role in influencing bacterial attachment to the alloy surfaces. The study conducted by Mu et al.^26^ reveals that surface roughness significantly influences bacterial adhesion, demonstrating that variations in roughness can lead to a 75-fold difference in bacterial fouling on hydrophobic surfaces. Specifically, increasing surface roughness generally enhances bacterial adhesion due to a larger effective surface area and decreased activation energy required for bacteria to attach. Additionally, wetting properties, such as the water contact angle, could play a significant^27^ role in determining the antibacterial efficacy of the CuAgZr alloys. Surface hydrophilicity or hydrophobicity can influence bacterial adhesion, as hydrophobic surfaces tend to promote the attachment of certain bacteria.^26,28^ While this study did not evaluate the wetting properties of the alloys, we recognize that this factor could be critical for understanding the broader antibacterial behavior of the materials.

The amorphous structure not only provides a smoother surface which may prevent bacterial adhesion^29^ but also offers a homogeneous microstructure and element distribution (Figures 1b and S4). This homogeneity is advantageous for predicting long-term alloy properties, such as in corrosive environments that involve microbial activity or the use of cleaning and disinfecting agents. Amorphous alloys, or metallic glasses, are known for their disordered atomic arrangement, which differs significantly from the crystalline structure of traditional alloys. This lack of long-range order results in unique properties, such as enhanced corrosion resistance compared to their crystalline counterparts. For instance Naka et al.^30^ investigated Cu_50_Ti_50_ and Cu_50_Zr_50_ alloys and found that in amorphous form their corrosion resistance was higher compared to crystalline alloys of the same composition. However, it is important to note that an amorphous structure does not uniformly equate to homogeneity in microstructure or elemental distribution. Amorphous alloys can exhibit compositional and topological short-range ordering, which can influence their physical and chemical properties. Variations in local atomic packing density and the presence of different atomic motifs can affect the material’s response to external stimuli, such as mechanical stress or chemical exposure. These characteristics are highly contingent on the chemical composition and the conditions of the synthesis process, as explored in our prior studies.^19,20,31^

Combinatorial synthesis via the PVD method offers the benefit of maintaining nearly identical processing conditions for alloys with varying chemical compositions. As a result, differences in the properties of the alloys will primarily depend on their chemical composition, which, under the given synthesis conditions, will determine the structure along with the type and quantity of defects and impurities. Analyzing the morphology of the produced films (Figures 1b and S4), it can be observed that different alloys exhibit varying tendencies to form cracks. The most cracks were observed in the zirconium-enriched areas. The film quality (Figure S4) significantly impacts the mechanical properties (Figure 1c). Indeed, in our recent work^19^ comparing the impact of DCMS and high power impulse magnetron sputtering (HiPIMS) on the structure and properties of alloys from the same system, i.e., CuAgZr, it was noted that HiPIMS allowed for up to 44% increase in hardness for certain compositions, which was explained by the improved quality of the coatings, including a reduced number of defects (e.g., no cracks were found). An important observation is that despite the presence of some imperfections in the films, it was possible to identify alloys with very high hardness. Figure 1c indicates the region where the hardness exceeds 5 GPa, comparable to the hardness of some martensitic stainless steels (e.g., AISI 420A) used in manufacturing surgical cutting instruments like scissors, scalpels, and knives.^8^ It is also worth noting that in hospitals, where infections are common, a considerable number of metal furniture is made from austenitic stainless steel, with a hardness of about 2 GPa (for AISI 316L).^32^

When considering the results of antibacterial tests a particularly important observation is the excellent reproducibility of results, despite tests being conducted on three separately produced CuAgZr MatLibs (i.e., synthesized in three runs of PVD). This demonstrates that the methodology utilizing thin-film MatLibs can provide highly reliable results regarding antibacterial activity. Furthermore, it allows for the precise identification of the boundary separating alloys with antibacterial properties from those that do not exhibit such characteristics.

Adjusting elemental proportions in the CuAgZr system can result in materials that not only maintain mechanical strength required for medical device applications but also exhibit bactericidal properties. In the realm of biomedical engineering, the selection of material for device fabrication is not only dictated by performance but also by cost efficiency. This delicate balance is illustrated on the CuAgZr ternary diagram, which maps out the material cost, determined here based on the expenses of the materials used for synthesizing the MatLibs examined in this study (Figure 1e). Alloys situated in the highlighted “Similar properties” zone present an optimal blend of amorphous structure, characterized by smooth surface and homogeneous distribution of elements, high hardness, exceeding 5 GPa, akin to that of certain martensitic stainless steels,^8^ while also demonstrating substantial antibacterial activity. The economic advantage of these alloys becomes evident when considering the cost of raw materials. Alloys with a higher content of silver, while offering certain performance merits, are overshadowed by the cost-effectiveness of those with increased proportions of copper, which exhibit a similar or superior performance profile. In clinical environments, where the battle against microbial contamination is relentless, the use of these cost-effective alloys could lead to significant budgetary alleviations. By coating traditional materials, such as austenitic stainless steel with hardness around 2 GPa,^32^ with CuAgZr alloy films with hardness of >5 GPa, a double gain can be expected: enhancement in surface scratch resistance and the innate antibacterial efficacy conferred by the copper and silver components.

The current discourse in experimental high-throughput materials science, as illustrated through the CuAgZr alloy system, is not a declaration of an ultimate solution but rather an exploration into the realm of potential sustainable antibacterial surfaces. The CuAgZr system serves as a model, its selection founded on the bactericidal properties of copper and silver, and the tendency of zirconium to increase glass forming ability in system with copper and silver.^12,33−35^ This research underscores the potential of CuAgZr alloys for biomedical use, yet it also recognizes the need for comprehensive validation. It is crucial to not only confirm their efficacy in retaining antibacterial properties after repeated sterilization procedures and their resilience to mechanical wear but also to assess the biocompatibility and potential toxicity of these materials in a scenario in which these alloys would be considered suitable for implants. While promising in bacterial studies, the safety profile of CuAgZr alloys must be thoroughly evaluated to ensure they pose no risk to healthcare environments before they can be considered suitable for hospital use.

The versatility of CuAgZr alloys extends to a variety of applications from the tools in the hands of surgeons to the textiles that clothe medical professionals and the surfaces within operating rooms (Figure 1f). The idea of using metallic glasses with antibacterial properties in medicine is not new and has been proposed by other authors for both surgical tools^36^ and textiles.^37^ The novelty of this work lies in demonstrating that high-throughput materials science enables the efficient selection and optimization of materials with desired properties, including targeted antibacterial activity. It should also be noted that the best alloys can be produced in bulk for entire tools, implants, and furnishings in operating rooms, indicating that the choice of materials utilizing this technology is not confined to mere surface treatments. Caution must be exercised, however, due to the fact that the synthesis process can in some way affect the desired properties,^38^ such as mechanical ones, as we demonstrated in previous work comparing thin-film metallic glasses with bulk counterparts produced by arc melting.^31^ Nonetheless, the advancement of PVD synthesis technology, allowing access to a wide range of energy states and control of impurities in the material, offers hope that differences in properties between thin-film materials and those produced using other methods, e.g., arc melting, can be mitigated.^19,39−41^

## Conclusion

This study demonstrated the application of high-throughput combinatorial synthesis and characterization to rapidly screen and identify CuAgZr alloys with both high mechanical performance and antibacterial efficacy.

XRD analysis revealed that crystalline regions with a FCC structure are predominant near the Cu–Ag edge, while amorphous regions increase toward the Zr-rich areas, particularly along the Cu–Zr edge. The amorphous structure provides a smoother surface with a homogeneous microstructure and uniform elemental distribution, as confirmed by EDX analysis.

Nanoindentation tests revealed that the hardness of the investigated CuAgZr alloys varies significantly with chemical composition, ranging from 2.2 to 5.7 GPa, with the hardest alloys being copper-rich and the softest enriched in zirconium. Despite zirconium’s high melting point, its presence led to increased cracking in the films, negatively impacting the hardness and mechanical properties of the alloys.

The antibacterial tests showed that CuAgZr alloys with higher copper and silver content exhibited strong antibacterial properties against *S. aureus*, while zirconium-rich alloys were less effective. The methodology ensures reliable reproduction of advantageous alloy compositions, allowing for rapid identification of bactericidal and nonbactericidal alloys. Ion release measurements confirmed that antibacterial activity was linked to copper ion release, though the role of silver remains unclear.

These findings suggest that CuAgZr alloys can be optimized for biomedical applications, offering both durability and strong antibacterial performance. The materials show potential for use as coatings in hospital environments, such as surgical instruments and operating room surfaces. The results highlight the need for continued exploration of CuAgZr or other alloy systems for sustainable antibacterial applications in healthcare. Further investigation into the long-term stability of CuAgZr alloys, including their resistance to repeated sterilization and mechanical wear, is essential. Additionally, future studies should aim to clarify the role of silver in the antibacterial behavior of these alloys and explore their biocompatibility for broader medical applications.

## Experimental Section

### Material Synthesis

Thin-film ternary CuAgZr MatLibs were fabricated in a vacuum chamber (Korvus Technology, U.K.) using the PVD technique, specifically DCMS, onto a 4-in. (100)-oriented silicon substrate, coated with a 100-nm-thick amorphous silicon nitride layer (Figure S2). The cosputtering process utilized high-purity targets of Cu, Ag, and Zr (sourced from HMW Hauner GmbH with a purity of 99.99%), which created a compositional gradient across the surface of the wafer. The sputtering process took place in a chamber fitted with a combination of rotary and turbo pumps, achieving a high base vacuum around 6 × 10^–7^ mbar. Argon gas with a purity of 99.9999% was used for cosputtering at a working pressure of 1 × 10^–2^ mbar. The CuAgZr MatLibs were crafted into 21 discrete circular patches, each with a diameter of 5 mm, using a stainless-steel mask that was 2 mm thick. The distance between the substrate and the target was finely tuned to produce the intended large compositional gradient on the wafer. The deposition powers for Cu, Ag, and Zr were set at 27, 19, and 75 W respectively. The deposition rate was between 10.6 and 18.6 nm per minute depending on the position on the wafer. In total, 7 MatLibs were synthesized. One of these was utilized for the investigation of structural properties using XRD, mechanical properties by nanoindentation, and surface morphology using SEM. Three of the MatLibs were designated for the study of antibacterial properties, and selected patches from the remaining three were used for ion release studies.

### Chemical Composition and Thickness

The chemical composition and thickness of the deposited MatLibs were ascertained through X-ray fluorescence (XRF) spectrometry (Fischerscope X-ray XDV-SDD by Fischer, Germany). An analytical beam with an energy setting of 50 kV and a spot size of ∼0.3 mm in diameter was employed for the measurements (the outcomes of which are compiled in Tables S1–S3). Due to the presence of a compositional gradient within each patch, efforts were made to characterize this variance. This is crucial because the antibacterial properties and ion release information determined in the experiments of this study are derived from the surface of each patch, which represents a range of chemical compositions, rather than from a single point with a precisely defined chemical makeup. The XRD experiment, on the other hand, was designed to collect crystallographic information from the entire volume of the 5-mm-diameter patch. The method used to determine the chemical composition gradient began by identifying both the central position of each patch and the content of each element at the center of each patch using XRF methodology. A two-dimensional cubic function was then employed to define the surfaces of chemical composition, representing the elemental content as a function of the *x* and *y* positions. Using these surfaces, the gradient for each element within the patches was calculated. This methodology provided a systematic approach to assessing the direction of elemental content changes in each section of the studied MatLibs. It should be noted that the fit of the function, represented by the *R*^2^ value, was greater than 0.99 in most cases, indicating a high degree of accuracy of the model. Visualization of the chemical composition and thickness gradient within each patch of the representative CuAgZr MatLib1 is shown in Figure S2.

To estimate the diversity of alloy compositions on a 4-in. silicon wafer, we employed a model predicated on the simplification of a linear compositional gradient of 65%. This simplification was instrumental in reducing computational complexity. We defined distinct alloys under the criterion that the sum of differences in elemental compositions between any two points on the wafer surface equals 1%. For the analysis of the ternary system, we posited that a 0.33% change in the concentration of any given element between two alloys occurs every 51.28 μm. This specification was crucial in identifying unique points on the wafer, where each of the three elements contributed equally to the overall 1% difference in composition, thereby delineating distinct alloys. By calculating the total area of the wafer against the area represented by each unique compositional shift of 0.33% per element, we determined the number of distinct alloys to be 29 865.

### Phase Identification

To determine the structure of investigated alloys, patches within representative CuAgZr MatLib were measured using XRD. The measurements were carried out with Cu Kα_1_ and Cu Kα_2_ radiation (λ = 1.5406 and 1.54439 Å, respectively) by means of a D8 Discover diffractometer (Bruker, Billerica, MA), equipped with a programmable sample-positioning stage. The phase composition of the CuAgZr MatLibs was identified using XRD data in the 2θ range from 30 to 90° obtained under the conditions: voltage of 40 kV, current of 40 mA, step size of 0.02°, and collection time at each step of 2 s. The θ/2θ scans were performed with an offset of −4° from the symmetrical diffraction geometry to avoid a too high intensity from the (400) reflection of the (100)-oriented single-crystal Si substrate. X-ray diffractograms showing peak locations as a function of 2θ angles are additionally summarized in Figure S3.

### Microstructural Investigations

Microstructural observations of top views of CuAgZr MatLib1 were performed at the center of each of the 21 patches using a Hitachi S-4800 (Chiyoda, Japan) cold field-emission high-resolution SEM. The morphology of each of the CuAgZr MatLib1 patches is summarized in Figure S4. The elemental distribution within the microstructure of selected Cu_34.5_Ag_27.8_Zr_37.7_ alloy (sample 11 of MatLib1) was examined using a Themis 200 G3 transmission electron microscope (Thermo Fischer, Waltham, MA) with spherical aberration correction, operating at 200 kV. For transmission electron spectroscopy (TEM) analysis, a lamella was prepared through focused-ion-beam milling (FIB technique) using a Tescan Lyra dual beam system (Brno, Czech Republic). Elemental analysis was conducted with EDX.

### Nanoindentation Tests

Nanoindentation experiments were conducted using a ZHN Nanoindenter by Advanced Surface Mechanics GmbH (Germany), equipped with a Berkovich diamond tip. The hardness and elastic modulus were determined using the Oliver–Pharr method.^42^ Prior to measurements, the tip area function and instrument compliance were calibrated for the appropriate measurement range using SiO_2_ (*E* = 72 GPa; ν = 0.17) and Al_2_O_3_ (*E* = 410 GPa; ν = 0.234) as described in detail in ref (43). The experiments on CuAgZr MatLib utilized the quasi-continuous stiffness measurement (QCSM) method. QCSM is a module developed by ASMEC to enable the determination of the contact stiffness of the sample not only based on the unloading curve at a single depth but at multiple points during the indentation process. This allows for depth-dependent determination of hardness and Young’s modulus at the same sample location. On each of the examined patches of the representative CuAgZr MatLib, 12 measurements were performed with a force reaching up to 100 mN. The measurements were conducted near the center of the patches to easily correlate them with the chemical composition determined by the XRF method. Indentations were spaced at an interval of 50 μm to ensure the suggested indentation depth/spacing ratio of 10 was maintained^44^ for the applied load. The averaged QCSM curves for hardness and reduced modulus are included in Figure S5. The hardness results presented in the paper were determined for indentation depths ranging from 250 to 500 nm.

### Antibacterial Activity

In this study, we used colony counting to evaluate the antibacterial properties of CuAgZr MatLibs against *S. aureus*, a common pathogenic bacterium. Our experimental design was structured to ensure reproducibility and accuracy. Details of the bacterial strains and media used in this study are summarized in Table S4.

Initially, for preculture a single colony of *S. aureus* was selected and introduced to 25 mL of 30% Tryptic Soy Broth (TSB) supplemented with 0.25% glucose. This mixture was then incubated overnight at 37 °C with a constant agitation of 160 rpm.

Upon completion of the overnight incubation, the optical density (OD) of the preculture was measured at wavelength 600 nm (OD600 nm). This culture was then diluted to achieve an OD600 nm of 0.1 in a fresh 25 mL of 30% TSB with 0.25% glucose. The diluted culture was further incubated for 1.5 h under the same conditions to reach the exponential growth phase, which is a critical stage for testing antibacterial activity. Subsequently, the OD600 nm of this exponentially grown culture was measured and further diluted to an OD600 nm of 0.05 using 1× phosphate-buffered saline (PBS). This step is pivotal to standardize the bacterial concentration for application onto the silicon wafer samples.

Following the dilution, 50 μL of the bacterial suspension was meticulously applied to the patches of the CuAgZr MatLibs and left for a 2-h interaction period. Postexposure, the bacterial suspension was carefully removed from the CuAgZr MatLibs and from a serial dilution of 1:10 20 μL were transferred to the agar plates. The agar plates were incubated for 18 h at 37 °C without agitation.

The observation and documentation included photographic records and colony counting, allowing quantification of the antibacterial effect of alloys from the CuAgZr MatLibs.

### Ion Release Tests

An ion release examination was conducted with the objective of gaining a better understanding of the causes behind the antibacterial properties of some of the examined alloys, namely, whether the release of individual ions is correlated with antibacterial properties. This investigation is crucial for understanding the mechanisms of antibacterial action in these materials, which may have significant implications for future biomedical and material engineering applications.

The release experiment was conducted using ICP-OES to evaluate the leaching properties of selected samples. These samples, specifically patches from the MatLib series 5, 6, and 7, underwent this analysis. Each sample was immersed in 3 mL of deionized water for a period of 4 h, facilitating ion leaching into the aqueous medium. Subsequently, the leachate was mixed with 3 mL of nitric acid (HNO_3_) and subjected to microwave heating for digestion, ensuring a comprehensive release of ions from the sample matrix. Postdigestion, the solutions were transferred to 15 mL plastic containers, readying them for ICP-OES analysis. The chemical compositions and results of ICP-OES experiments of the analyzed samples are detailed in Table S3 and Figure S6.

While this method provides initial insights into the leaching behavior of the alloys, it is important to acknowledge that the results obtained with deionized water may not directly correlate with those in bacterial studies.

## Supporting Information Available

Marking of patches and the appearance of the representative as-deposited CuAgZr MatLib1, visualization of the chemical composition and thickness gradient within each patch of CuAgZr MatLib1, X-ray diffractograms reflecting phase composition and amorphous/crystalline structure, thin-film morphology of CuAgZr MatLib1, mechanical properties of CuAgZr MatLib1 vs indentation depth, ICP-OES results for ion release, thickness and chemical composition of CuAgZr MatLibs used for structural, mechanical, antibacterial, and ion release studies, and bacterial strain used in the study (PDF)
