# Supplementary material for: Developing a High-Throughput Platform for the Discovery of Sustainable Antibacterial Materials
Source: ACS Appl Mater Interfaces. 2024 Oct 25;16(44):60018–26. doi: 10.1021/acsami.4c14689 (PMC11551899; doi:10.1021/acsami.4c14689)
Supplement: Supplementary file 1 — am4c14689_si_001.pdf [file am4c14689_si_001.pdf]

Supporting Information

Developing a high-throughput platform for the discovery of sustainable antibacterial materials

Krzysztof Wieczerzak[1], Fedor F. Klimashin[1], Amit Sharma[1], Stefanie Altenried[2], Katharina Maniura-Weber[2], Qun Ren[2], and Johann Michler[1]*

AUTHOR ADDRESSES

[1]Empa, Swiss Federal Laboratories for Materials Science and Technology, Laboratory for Mechanics of Materials and Nanostructures, CH-3602 Thun, Switzerland;

[2]Empa, Laboratory for Biointerfaces, Swiss Federal Laboratories for Materials Science and Technology, Lerchenfeldstrasse 5, CH-9014 St. Gallen, Switzerland;

*Corresponding Author**

*Krzysztof Wieczerzak – orcid.org/0000-0001-9976-7591;

Email: krzysztof.wieczerzak@empa.ch

Fedor F. Klimashin – orcid.org/0000-0003-0425-246X

Amit Sharma – orcid.org/0000-0002-9877-2601

Stefanie Altenried – orcid.org/0000-0001-7895-3563

Katharina Maniura-Weber – orcid.org/0000-0001-7895-3563

Qun Ren – orcid.org/0000-0003-0627-761X

Johann Michler – orcid.org/0000-0001-8860-4068

Supporting Information

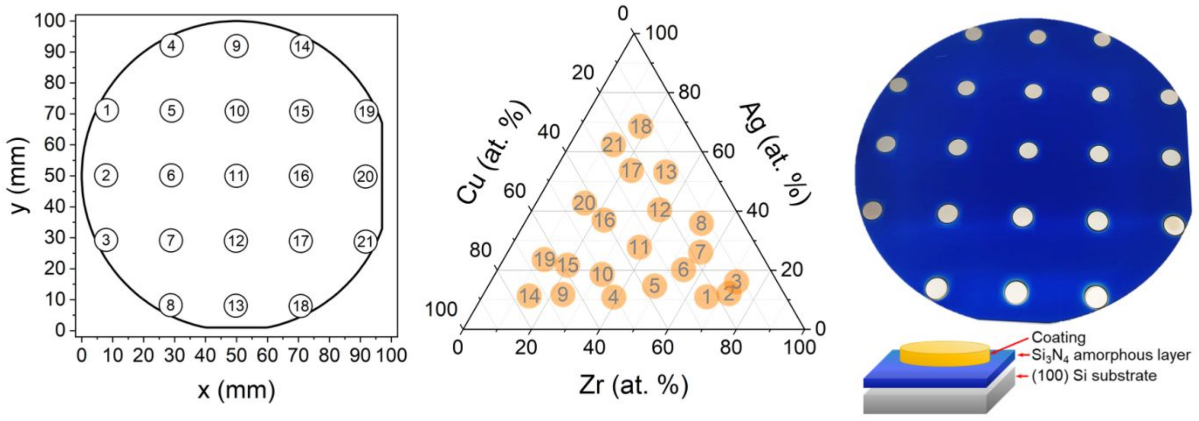

Figure S1 | Marking of patches and the appearance of the representative as-deposited CuAgZr MatLib1.

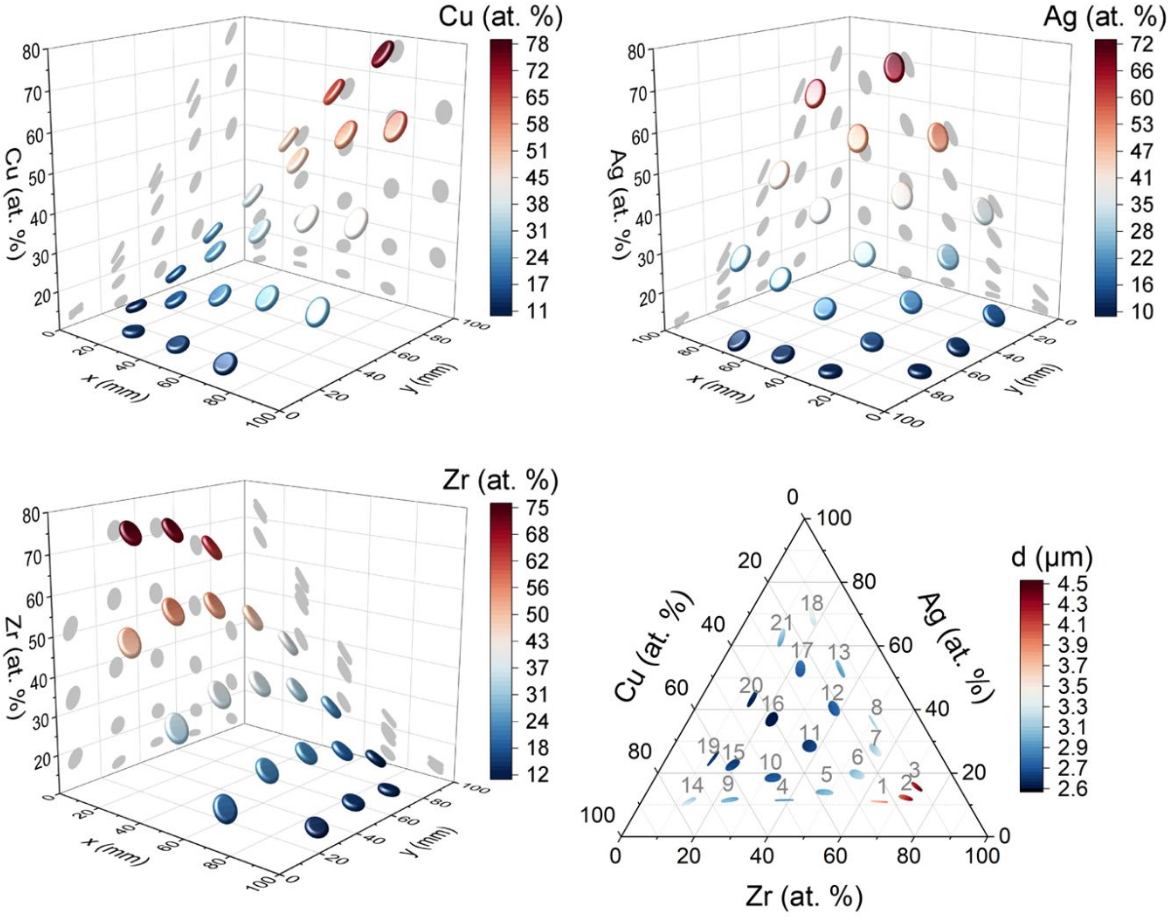

Figure S2 | Visualization of the chemical composition and thickness gradient within each patch of the representative CuAgZr MatLib1. The chemical composition of these patches is also given in Table S1. Regions exhibiting a high concentration of a specific element were found near the respective magnetron. In these regions, a marked concentration gradient is noticeable. This gradient diminishes progressively with increasing distance from the magnetron, eventually transitioning into a nearly uniform distribution.

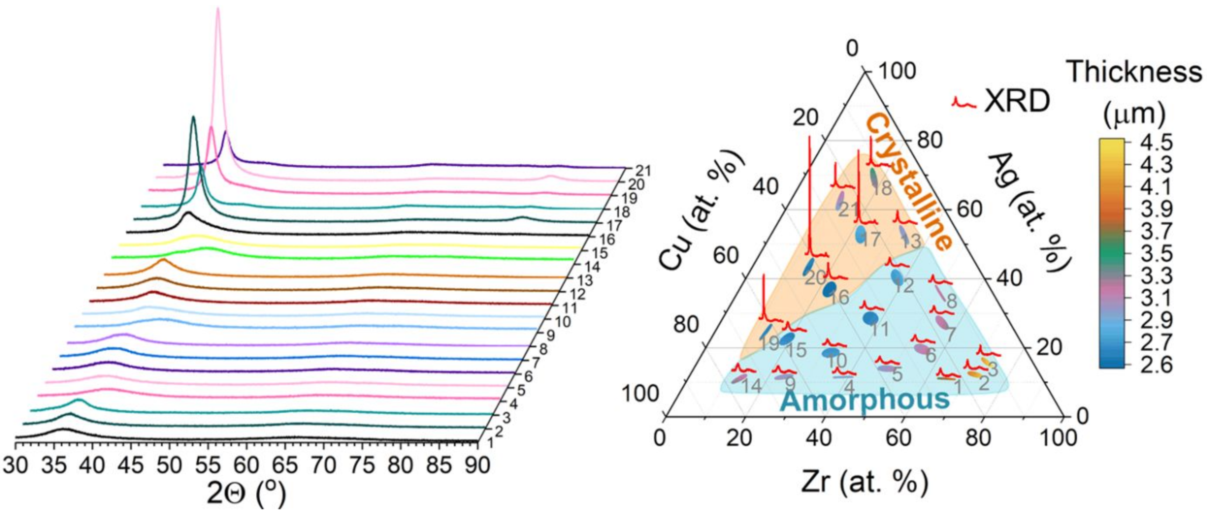

Figure S3 | X-ray diffractograms of patches of CuAgZr MatLib1. These diffractograms reflect the phase composition in each sample, where distinct peaks indicate crystallinity and the absence or broadening of peaks suggests an amorphous structure. The chemical composition of these patches is given in Table S1.

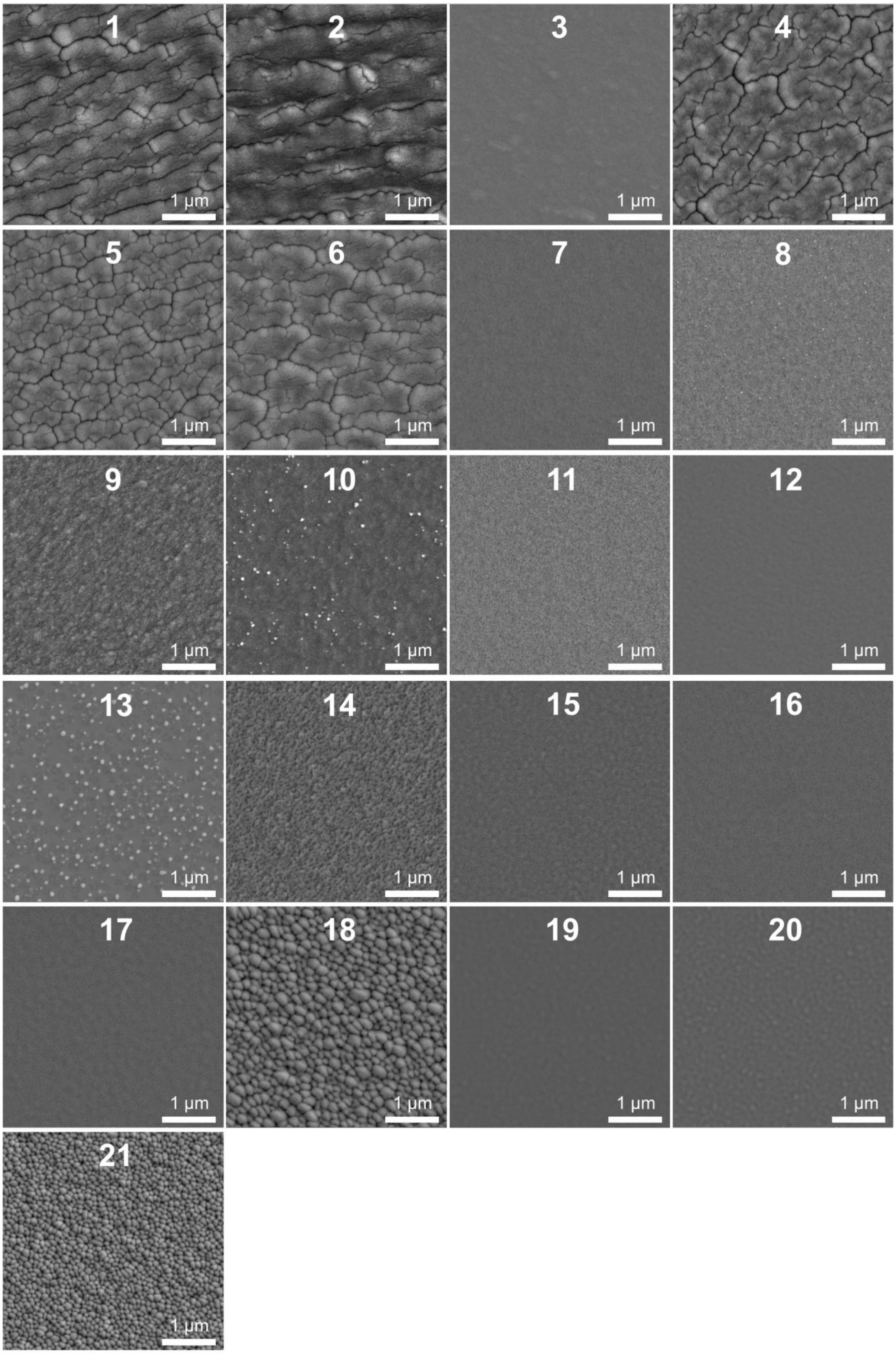

Figure S4 | The CuAgZr MatLib1 thin film morphology (top view). Images were taken at the center of each patch.

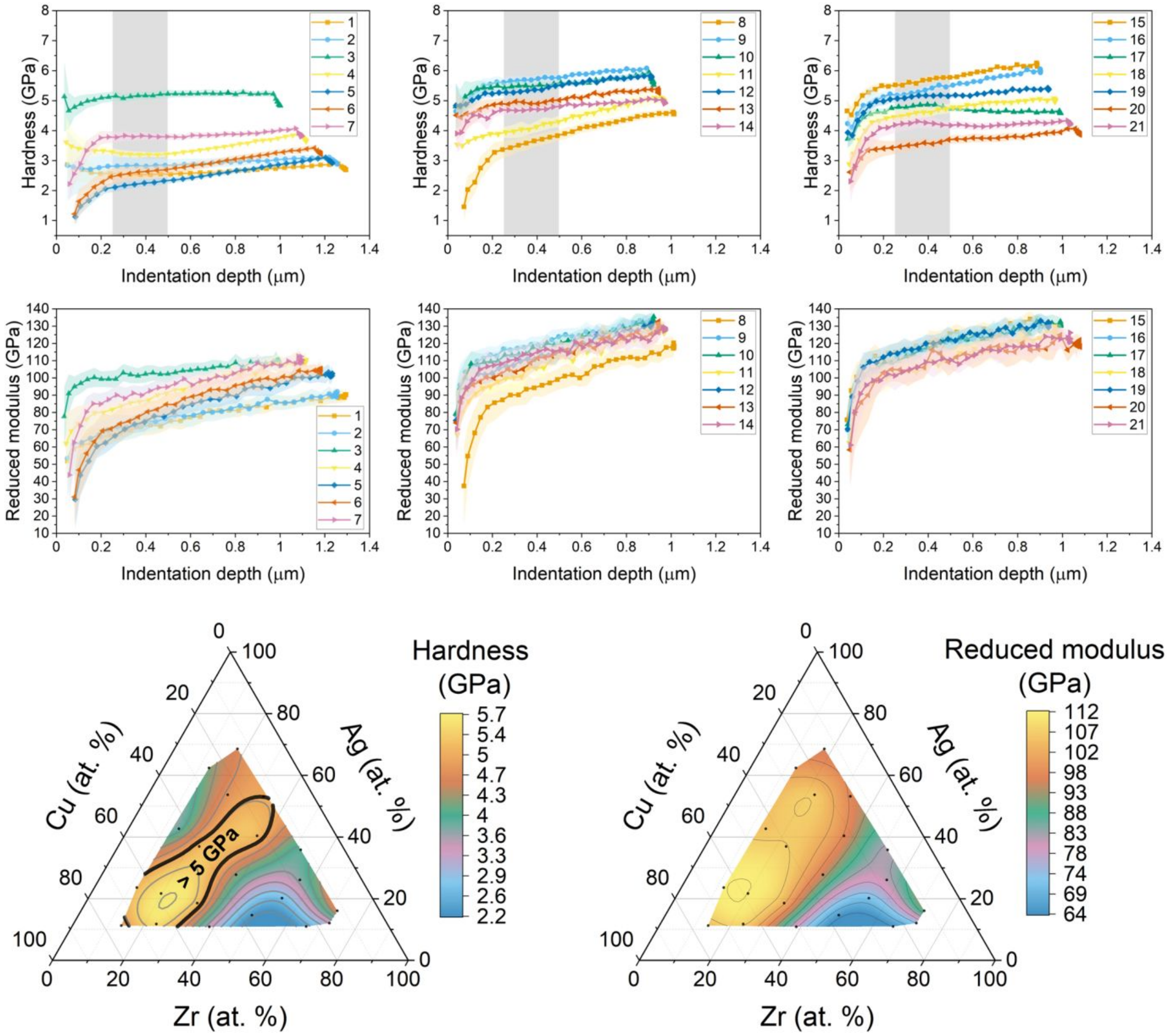

Figure S5 | Mechanical properties of the representative CuAgZr MatLib vs indentation depth. *The average values and standard deviations of hardness were determined based on data points in the range of indentation depth between 250 and 500 nm, marked with gray rectangles. Reduced modulus was obtained by extrapolation the linear fit to the data points above 250 nm back to zero indentation depth.*

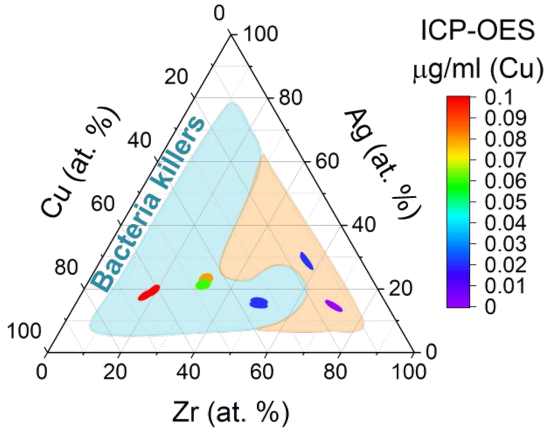

Figure S6 | Graphical representation of the ICP-OES results. Note that only Cu ions were detected.

Table S1. Thickness and chemical composition of the CuAgZr MatLib (1) used for the investigation of structural properties using XRD, mechanical properties by nanoindentation, and surface morphology using SEM. In the first row, for each of the 21 samples, the results measured directly by the XRF method at the center of each patch were placed, while in the second row, the calculated thickness gradient and the gradient of elemental content in the alloy for each of the 5 mm diameter patches were presented. Nanoindentation results (mean ± standard deviation) measured at the center of each of the 21 patches.

			MatLib 1			
**No**	d (μm)	Cu (at. %)	Ag (at. %)	Zr (at. %)	H (GPa)	Er (GPa)
**1**	3.72	23.2	11	65.8	2.6 ± 0.1	70.9 ± 4.8
	3.66-3.92	22-26.1	10.8-11.1	62.8-67.1		
**2**	4.22	16.1	12.1	71.8	2.8 ± 0.3	73 ± 7
	3.99-4.31	14.8-17.7	11.5-13	69.6-73.5		
**3**	4.31	11.9	16.1	72	5.1 ± 0.2	100.6 ± 4.9
	4.12-4.47	10.7-12.3	14.5-17	71-74.7		
**4**	3.05	50.4	10.9	38.6	3.2 ± 0.1	85 ± 4.4
	2.98-3.01	47.4-52.1	11.4-11.6	36.4-41.1		
**5**	3.07	36.6	14.7	48.7	2.2 ± 0.1	74.4 ± 5.3
	3-3.12	35.4-39.6	13.3-14.7	46.3-50.8		
**6**	3.12	25.4	20.2	54.4	2.6 ± 0.2	77.6 ± 6.9
	3.07-3.24	24.2-27.6	18.3-20.9	52.4-56.7		
**7**	3.21	17.4	26.1	56.5	3.8 ± 0.2	89.2 ± 5.3
	3.14-3.31	16.1-18.1	25.5-29.1	53.6-57.9		
**8**	3.17	12.3	35.9	51.9	3.5 ± 0.2	91 ± 7.3
	3.15-3.28	13.1-13.4	33.6-38	48.8-53.4		
**9**	3.03	64.9	11.8	23.3	5.6 ± 0.2	115 ± 5.1
	2.99-3.1	62.3-67	11-12.2	21.9-25.8		
**10**	2.73	50	18.6	31.4	5.5 ± 0.2	111.7 ± 5.1
	2.72-2.77	47.1-51.6	17.4-19.7	30.2-34.1		
**11**	2.7	34.5	27.8	37.7	4 ± 2.2	100.8 ± 6.2
	2.65-2.72	32.5-36.3	26.8-30.2	35.2-39.2		
**12**	2.78	22.3	40.4	37.3	5.3 ± 0.1	106.4 ± 4.9
	2.76-2.82	20.5-23.2	38.2-42.5	35.9-40		
**13**	3.02	14.1	53.2	32.7	4.9 ± 0.2	103.6 ± 6
	2.98-3.05	13.3-14.5	50.2-55.3	31.2-35.7		
**14**	3.2	75	11.3	13.7	4.6 ± 0.2	110 ± 6.6
	3.15-3.34	73.7-78.3	9.8-12.2	11.7-14.2		
**15**	2.69	58.6	21.7	19.7	5.5 ± 0.1	114.9 ± 4.8
	2.65-2.73	56-60.7	21-24.2	17.8-20.6		
**16**	2.56	40.3	37	22.8	5.2 ± 0.2	111.4 ± 6.6
	2.56-2.58	38.4-42.6	34.9-38.9	21.3-24.2		
**17**	2.81	23.9	53.7	22.4	4.7 ± 0.1	110.9 ± 4.6
	2.76-2.86	23.1-26.4	50.5-55.2	20.9-24.1		
**18**	3.4	13.7	68.6	17.7	4.5 ± 0.2	102.6 ± 6.8
	3.26-3.47	12.2-14.3	66.5-71.9	15.8-19.5		
**19**	2.72	64.4	23.6	11.9	5.1 ± 0.1	114.1 ± 6
	2.63-2.72	60.2-65	22.4-26.7	12.4-13.3		
**20**	2.63	43.1	42.7	14.2	3.5 ± 0.2	106.5 ± 7.7
	2.59-2.65	40.2-44.8	41-45.8	13.6-14.7		
**21**	3.02	24.8	62.4	12.8	4.2 ± 0.2	103.7 ± 7.2
	2.99-3.18	23-27	60-65.1	11.6-13.4		

Table S2. Thickness and chemical composition of the CuAgZr MatLibs (2-4) used for the investigation of antibacterial properties. In the first row, for each of the 21 samples, the results measured directly by the XRF method at the center of each patch were placed, while in the second row, the calculated thickness gradient and the gradient of elemental content in the alloy for each of the 5 mm diameter patches were presented. In the last column of Table S2, the results of the antibacterial tests are included, measured in the unit of colony forming units per milliliter (CFU/ml).

	**MatLib 2**				***S. aureus***
**No**	d (μm)	Cu (at. %)	Ag (at. %)	Zr (at. %)	CFU/ml
**1**	3.01	23.4	10.9	65.7	0.00E+00
	2.89-3.11	22.1-26.1	10.5-10.8	63.3-67.7	
**2**	3.2	15.9	11.3	72.8	4.55E+01
	3.08-3.33	14.5-17.4	11-12.4	70.1-74.1	
**3**	3.25	11.3	15.8	73	2.73E+04
	3.12-3.37	10.6-12	13.9-16.3	71.7-75.5	
**4**	2.3	50.5	10.3	39.2	0.00E+00
	2.29-2.34	47.5-52.4	11.1-11.3	36.7-41.4	
**5**	2.37	35.8	14.8	49.4	0.00E+00
	2.29-2.4	35.1-39.4	12.8-14.1	46.9-51.4	
**6**	2.38	25.5	19.2	55.3	0.00E+00
	2.34-2.48	23.9-27.3	17.7-20.2	53-57.3	
**7**	2.43	17.7	25.5	56.8	9.09E+01
	2.38-2.51	15.9-18	24.7-28.2	54.3-58.6	
**8**	2.4	12.2	34.8	53	4.77E+03
	2.35-2.45	12.9-13.2	32.7-37	50.2-54.7	
**9**	2.25	65.3	11.1	23.7	0.00E+00
	2.21-2.29	62.6-67.4	10.3-11.5	22.1-26.1	
**10**	2.06	50.1	17.7	32.2	0.00E+00
	2.02-2.07	47.4-51.9	16.6-18.9	30.8-34.8	
**11**	2.03	34.9	27.4	37.6	0.00E+00
	1.99-2.04	32.9-36.8	25.9-29.2	36-39.9	
**12**	2.11	22.6	39	38.5	2.05E+03
	2.07-2.12	20.9-23.7	37.1-41.4	36.8-40.9	
**13**	2.23	14.1	51.5	34.3	1.36E+02
	2.22-2.26	13.2-14.6	49.1-54.1	32.6-37	
**14**	2.37	76.1	10.5	13.5	0.00E+00
	2.31-2.45	74.5-79.2	8.5-10.9	11.7-14.5	
**15**	1.98	59.7	20.3	20	0.00E+00
	1.96-2.01	57-61.6	19.7-22.8	18.5-21.3	
**16**	1.85	41.8	33.7	24.4	0.00E+00
	1.89-1.91	39.5-43.7	33.6-37.6	22.1-25	
**17**	2.12	24	52.7	23.2	0.00E+00
	2.04-2.11	23.8-27.3	49.2-54	21.9-25.1	
**18**	2.44	13.8	67.1	19.1	0.00E+00
	2.39-2.52	11.8-14.3	65.3-70.7	17.1-20.8	
**19**	2.05	64.5	22.8	12.7	0.00E+00
	1.99-2.06	61-65.9	20.5-24.7	12.8-13.8	
**20**	1.93	44.7	40.6	14.8	0.00E+00
	1.93-1.97	40.9-45.6	39.3-44.1	14.3-15.5	
**21**	2.26	24.7	62	13.2	0.00E+00

	2.19-2.33	22.9-27.2	58.5-63.8	12.3-14.1	
			**MatLib 3**		***S. aureus***
**No**	d (μm)	Cu (at. %)	Ag (at. %)	Zr (at. %)	CFU/ml
**1**	3.03	22	10.2	67.8	8.64E+04
	2.91-3.14	20.9-24.7	7.8-9.2	64.7-68.7	
**2**	3.25	15.4	11.9	72.7	5.45E+02
	3.06-3.33	14.1-16.8	10.3-12.4	70.6-74.2	
**3**	3.19	11.6	15.8	72.6	2.86E+04
	3.1-3.39	10.8-12.1	13.5-16.4	71-74.5	
**4**	2.29	48.4	9.52	42.1	0.00E+00
	2.27-2.33	45.6-50.4	8.6-9.7	38.9-43.6	
**5**	2.34	35.1	14.3	50.6	0.00E+00
	2.32-2.42	33.9-38.2	13.1-14.9	48.8-53.2	
**6**	2.4	25	19	56	0.00E+00
	2.29-2.43	23.3-26.7	18.7-21.3	54.2-58.3	
**7**	2.36	17.3	25.5	57.2	3.50E+03
	2.28-2.42	15.8-17.8	25.5-28.9	54.5-58.6	
**8**	2.37	12.4	35.1	52.5	6.82E+04
	2.36-2.49	13.2-13.4	33.3-37.8	49.2-53.6	
**9**	2.25	64	10.8	25.2	0.00E+00
	2.23-2.27	61-65.8	9.7-11.4	23.7-27.9	
**10**	2.04	49.1	17.8	33.2	0.00E+00
	2.07-2.12	46.6-51.1	17.4-19.7	32.6-36.6	
**11**	2.04	34.1	26.9	38.9	0.00E+00
	1.96-2.01	32.5-36.4	26.6-29.6	37.3-41.2	
**12**	2.04	22.4	38.3	39.2	0.00E+00
	1.97-2.01	20.9-23.6	37.3-41.2	37.3-41.3	
**13**	2.2	14.4	52.1	33.5	0.00E+00
	2.17-2.25	13.5-14.8	49.5-54.5	32.1-36.5	
**14**	2.39	74.8	10.7	14.5	0.00E+00
	2.3-2.39	73.5-78	8.3-10.9	11.8-15	
**15**	1.99	59.4	20.5	20.1	0.00E+00
	2.01-2.07	56.7-61.3	19.7-22.7	19.4-22.4	
**16**	1.87	41.8	33.9	24.3	0.00E+00
	1.89-1.9	39.7-43.9	32.9-36.6	23-26	
**17**	2	25.2	51	23.9	0.00E+00
	1.96-2.03	24.3-27.8	47.9-52.5	22.4-25.6	
**18**	2.41	14.4	66.4	19.2	0.00E+00
	2.34-2.51	12.6-15	64.9-70.6	17-20.7	
**19**	2.02	65	21.7	13.2	0.00E+00
	1.98-2.04	61.5-66.2	19.6-23.6	12.5-13.8	
**20**	1.91	45.8	38.7	15.5	0.00E+00
	1.91-1.93	42-46.6	37.4-42	14.7-16	
**21**	2.18	25.8	60.4	13.8	0.00E+00
	2.11-2.24	24.3-28.6	56.4-61.8	12.9-14.7	

	MatLib 4				*S. aureus*
**No**	d (μm)	Cu (at. %)	Ag (at. %)	Zr (at. %)	CFU/ml
**1**	1.13	20.3	13.6	66	3.00E+04
	1.08-1.17	19.4-23	13.3-13.8	63.3-67.2	
**2**	1.18	14.7	15.2	70.1	1.73E+05
	1.12-1.21	13.2-15.6	14.2-15.7	68.9-72.5	
**3**	1.14	10.8	18.1	71.2	2.00E+05
	1.11-1.2	10.5-11.5	16.7-19.3	69.3-72.8	
**4**	0.834	46.7	12.6	40.7	0.00E+00
	0.83-0.86	43.8-48.8	12.6-13.2	38.5-43	
**5**	0.854	33.1	16.8	50	0.00E+00
	0.84-0.89	31.6-35.8	15.7-17.1	47.8-52	
**6**	0.874	23.2	21.6	55.3	0.00E+00
	0.85-0.9	21.6-24.7	20.8-23.2	52.8-56.9	
**7**	0.857	16.2	28.6	55.2	2.23E+05
	0.85-0.9	14.8-16.6	27.6-31	53.1-57.1	
**8**	0.874	11.7	38.2	50.1	5.91E+02
	0.85-0.88	12-12.3	35.7-40	47.9-52.3	
**9**	0.806	61.8	12.4	25.7	0.00E+00
	0.78-0.8	59.1-64.2	11.8-13.3	23.8-27.8	
**10**	0.731	46.4	20.4	33.1	0.00E+00
	0.73-0.75	43.8-48.3	18.9-21.1	32-35.9	
**11**	0.752	31.2	29.9	38.9	3.18E+02
	0.72-0.74	30.3-33.9	28.1-31.2	36.5-40.2	
**12**	0.759	21	40.9	38.1	0.00E+00
	0.75-0.76	19.5-22.1	39-43	36.4-40.3	
**13**	0.813	13	53.8	33.3	0.00E+00
	0.81-0.82	12.3-13.7	51.2-56	31.4-35.6	
**14**	0.827	73.2	12.4	14.4	0.00E+00
	0.81-0.85	71.4-76.3	10.8-13.2	12.8-15.7	
**15**	0.711	56.4	22	21.6	0.00E+00
	0.71-0.72	53.5-58	21.8-24.8	19.7-22.4	
**16**	0.679	38.7	37.3	24.1	0.00E+00
	0.68-0.69	36.9-40.9	34.9-38.7	23-25.8	
**17**	0.735	24.1	52.3	23.7	0.00E+00
	0.73-0.75	22.7-25.9	49.8-54.3	22.4-25.4	
**18**	0.876	13.1	67.9	19	0.00E+00
	0.86-0.91	11.6-13.9	65.9-71.1	17-20.5	
**19**	0.736	60.9	25.3	13.9	0.00E+00
	0.72-0.75	57.5-62.2	23.9-27.8	13.9-14.9	
**20**	0.699	41.7	41.8	16.5	0.00E+00
	0.69-0.71	38.4-42.8	41-45.4	15.8-16.8	
**21**	0.797	23.7	61.7	14.6	0.00E+00
	0.77-0.81	22.1-26	58.8-63.8	13.9-15.5	

Table S3. Thickness and chemical composition of selected patches of the CuAgZr MatLibs (5-7) used for the ion release studies with ICP-OES. In the first row, for each of the 21 samples, the results measured directly by the XRF method at the center of each patch were placed, while in the second row, the calculated thickness gradient and the gradient of elemental content in the alloy for each of the 5 mm diameter patches were presented. Note that only Cu ions were detected.

**MatLib5**					**ICP-OES results**				
**No**	d (μm)	Cu (at. %)	Ag (at. %)	Zr (at. %)	Emission λ	Reading (mg/l)	Average (ug/ml)	Dilution	Results (ug/ml)
**3**	3.1	15.7	15.5	68.8	Cu 324.754	0	0.00	2	0.00
	3-3.3	14.2-16.4	13.6-16.6	69-75	Cu 327.395	0			
**5**	2.57	32.7	15.8	51.5	Cu 324.754	0.01	0.01	2	0.02
	2.5-2.6	31.6-35.6	15-16.8	46.5-51.1	Cu 327.395	0.01			
**8**	2.31	15.4	39	45.7	Cu 324.754	0.01	0.01	2	0.02
	2.3-2.4	15.1-15.9	37.2-41.9	43.1-48.4	Cu 327.395	0.01			
**10**	2.17	45.5	23.1	31.3	Cu 324.754	0.04	0.04	2	0.08
	2.1-2.2	43.2-47.5	21.8-24.8	30.1-33.4	Cu 327.395	0.04			
**15**	2.23	63.5	19.4	17.1	Cu 324.754	0.05	0.05	2	0.10
	2.2-2.3	60.6-65.3	18.3-21.5	18.5-20.8	Cu 327.395	0.05			

**MatLib6**					**ICP-OES results**				
**No**	d (μm)	Cu (at. %)	Ag (at. %)	Zr (at. %)	Emission λ	Reading (mg/l)	Average (ug/ml)	Dilution	Results (ug/ml)
**3**	3.12	15.7	15.7	68.5	Cu 324.754	0	0.00	2	0.00
	3-3.4	13.3-15.9	13.4-16.1	69.1-74.6	Cu 327.395	0			
**5**	2.52	33.4	15.5	51.1	Cu 324.754	0.01	0.01	2	0.02
	2.4-2.5	32.7-36.9	14.1-15.9	47.4-52.1	Cu 327.395	0.01			
**8**	2.24	15.8	37.3	46.9	Cu 324.754	0.01	0.01	2	0.02
	2.2-2.3	15.4-16.4	35.2-39.8	42.5-47.4	Cu 327.395	0.01			
**10**	2.06	46.6	21.5	31.9	Cu 324.754	0.03	0.03	2	0.06
	2.1-2.1	45.2-49.5	20.2-23	30.3-33.8	Cu 327.395	0.03			
**15**	2.16	65.9	17.6	16.5	Cu 324.754	0.05	0.05	2	0.10
	2.2-2.3	62.8-67.6	16.8-19.7	16.6-19.4	Cu 327.395	0.05			

**MatLib7**					**ICP-OES results**				
**No**	d (μm)	Cu (at. %)	Ag (at. %)	Zr (at. %)	Emission λ	Reading (mg/l)	Average (ug/ml)	Dilution	Results (ug/ml)
**3**	3.14	15.8	15.1	69	Cu 324.754	0	0.00	2	0.00
	3-3.4	13.3-15.9	13.1-15.9	68.7-74.3	Cu 327.395	0			
**5**	2.54	34.3	14.8	50.9	Cu 324.754	0.01	0.01	2	0.02
	2.4-2.5	32.7-36.9	14-15.7	47.5-52.2	Cu 327.395	0.01			
**8**	2.21	16	36.8	47.2	Cu 324.754	0.01	0.01	2	0.02
	2.1-2.2	15.4-16.4	35.1-39.7	42-46.9	Cu 327.395	0.01			
**10**	2.09	47.3	21.2	31.5	Cu 324.754	0.03	0.03	2	0.06
	2.1-2.1	45.2-49.5	20.1-22.9	30.3-33.9	Cu 327.395	0.03			
**15**	2.2	66.1	17.7	16.2	Cu 324.754	0.05	0.05	2	0.10
	2.2-2.3	62.8-67.6	16.7-19.7	16.6-19.4	Cu 327.395	0.05			

Table S4. Bacteria strain used in the study Staphylococcus aureus ATCC 6538.

**Pre-culture:**	30% Tryptic Soy Broth (TSB) + 0.25% Glucose (Sigma-Aldrich)	
	Casein Peptone	* 17.0 g/L (* 100%)
	Soya peptone	* 3.0 g/L
	Sodium chloride	* 5.0 g/L
	Dipotassium hydrogen phosphate	* 2.5 g/L
	Glucose	* 2.5 g/L
	pH adjusted to neutral at 25 °C	
**Media:**	Plate Count Agar (Sigma-Aldrich)	
	Tryptone	5 g/L
	Yeast extract	2.5 g/L
	Dextrose	1 g/L
	Agar	12 g/L
	pH adjusted to neutral at 25 °C	
**PBS:**	Phosphate buffered saline (Sigma-Aldrich)	
